# Reversible ifosfamide-induced encephalopathy with bursts of triphasic waves responsive to levetiracetam

**DOI:** 10.1055/s-0044-1789267

**Published:** 2024-09-02

**Authors:** Luis Eduardo Borges de Macedo Zubko, Lucas Altoé Brandão, Caio Cesar Diniz Disserol, Igor Ibrahim Nascimento, Luciano de Paola

**Affiliations:** 1 Universidade Federal do Paraná, Complexo Hospital de Clínicas, Departamento de Neurologia, Curitiba PR, Brazil.; 2 Universidade Federal do Paraná, Complexo Hospital de Clínicas, Departamento de Neurofisiologia, Curitiba PR, Brazil.

A 42-year-old woman with a history of metastatic osteosarcoma and chronic kidney disease presented to our emergency department due to acute altered mental status that had started two days after chemotherapy infusion with ifosfamide and etoposide. Initially, she was alert, confused, and disoriented. On the third day, she developed echolalia, perseveration, and paroxysmal events of behavioral arrest. A brain magnetic resonance imaging (MRI) scan and the laboratory work up were unremarkable. An electroencephalogram (EEG) revealed diffuse slowing of background activity and bursts of triphasic waves ( Figure 1 ). The patient was managed with suspension of the ifosfamide and introduction of oral levetiracetam, which lead to improvements in the EEG and in cognition ( Figure 2 ). Reversible EEG changes and antiseizure-responsive encephalopathy may be observed during ifosfamide therapy. 1 2 3

**Figure 1:**
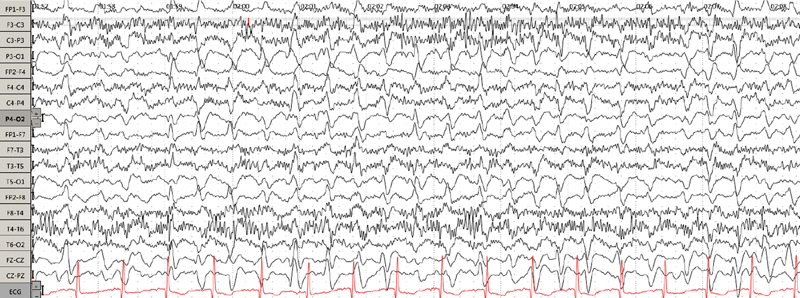
Three days after the onset of symptoms. Electroencephalogram (EEG) showing diffuse slowing of background activity and bursts of triphasic waves, with each burst lasting between 10 to 20 seconds.

**Figure 2:**
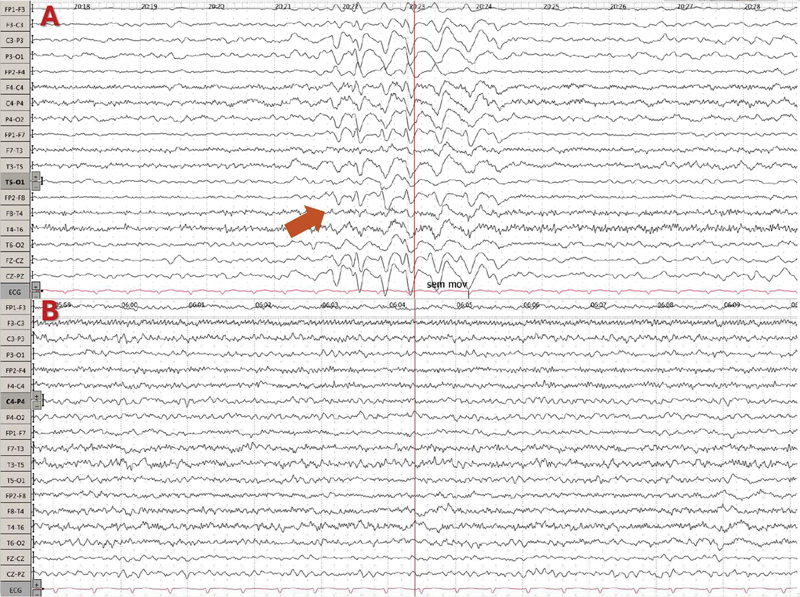
( **A** ) Four days after the onset of symptoms. Second EEG after 1 day of levetiracetam introduction, showing partial improvement. ( **B** ) Eight days after the onset of symptoms. Third EEG, showing complete resolution.

## Notes

Appendix A
**Quiz**

1. What are the predisposing factors for ifosfamide-induced encephalopathy?
  A) Renal insufficiency.
  B) Hepatic insufficiency.
  C) Higher doses.
  D) Poor functional status at treatment initiation.
  E) All of the above.
  Answer: E.
2. What is the main pathophysiology for the central nervous system associated with ifosfamide?
  A) A metabolic disturbance due to the depletion of glutathione caused by the toxic metabolite chloroacetaldehyde.
  B) Excitotoxicity due to increase in glutamate transmission.
  C) Neuroinflammation due to activation of microglia.
  D) Direct neurotoxicity due to disruption of ion channels.
  E) Disruption of the blood-brain barrier.
  Answer: A.
3. What other medications may be associated with triphasic waves on an electroencephalogram (EEG)?
  A) Lithium.
  B) Metronidazole.
  C) Pregabalin.
  D) Cefepime.
  E) All of the above.
  Answer: E.
4. How long does it usually take for the CNS symptoms to improve with treatment after an episode of ifosfamide-associated encephalopathy?
  A) 6 to 12 hours.
  B) 12 to 24 hours.
  C) 48 to 72 hours.
  D) 72 to 96 hours.
  E) None of the above.
  Answer: C.
